# Review of Covid-19 vaccine clinical trials - A puzzle with missing pieces

**DOI:** 10.7150/ijbs.59170

**Published:** 2021-04-10

**Authors:** Hang Fai Kwok

**Affiliations:** Institute of Translational Medicine, Faculty of Health Sciences, University of Macau, Avenida de Universidade, Taipa, Macau SAR

**Keywords:** SARS-CoV-2, Pandemic, Vaccination, Different subgroups, Viral Variants

## Abstract

A year after the initial outbreak of Covid-19 pandemic, several Phase III clinical trials investigating vaccine safety and efficacy have been published. These vaccine candidates were developed by different research groups and pharmaceutical companies with various vaccine technologies including mRNA, recombinant protein, adenoviral vector and inactivated virus-based platforms. Despite numerous successful clinical trials, participants enrolled in these trials are limited by trial inclusion and exclusion criteria, geographic location and viral outbreak situation. Many questions still remain, especially for specific subgroups, including the elderly, females with pregnancy and breastfeeding status, and adolescents. At the same time, vaccine efficacy towards asymptomatic infection and specific viral variants are still largely unknown. This review will cover vaccine candidates with Phase III clinical trial data released and discuss the scientific data available so far for these vaccine candidates for different subgroups of people and different viral variants.

## Introduction

The SARS-CoV-2 pandemic is a global crisis that has not yet been resolved. Currently, there are more than 100 million confirmed Covid-19 cases and the disease has claimed more than 2 million lives around the world. The implementation of strict measures in some countries or cities, such as widespread testing, isolation of infected individuals, and lockdown of part of the countries or region, along with the uncontrolled increasing number of cases, may have slowed down the infection. However, it does come with significant sociological, psychological and economic challenges. Not only do Covid-19 patients and front-line healthcare professionals have an increased risk of mental health problems resulting in a profound effect on their daily life 1, 2, but there is also a decrease in positive emotions and life satisfaction among the general population, as well as the elderly population that may impact their mental health resulting in an increased risk of development of psychiatric disorders 3, 4.

Like our history of success in combating highly infectious viruses to save a life, developing a highly efficacious and safe vaccine in which known risk is weighed against potential benefit is one of the most effective strategies 5. However, creating a successful vaccine candidate that is safe and efficacious for human administration requires an enormous effort. A particular challenge is the provision of long-lasting immunity, as the current evidence available suggests that humoral markers of immunity in patients infected with either the SARS or MERS-CoV were absent when they were re-tested 5-6 years later, while re-infection of SARS-CoV-2 has also been reported 6. Several seasonal human coronaviruses cause the common cold in humankind and two novel coronaviruses, SARS and MERS-CoV, that emerged in the past 18 years have overcome the species barrier to infect human. However, prior to the success of several Phase III trials of various vaccine candidates targeting SARS-CoV-2, there was no vaccine licensed or available for the other coronaviruses affecting human 6, 7.

The race to develop a vaccine targeted against SARS-CoV-2 has led to vaccine candidates based on different strategies, encompassing both traditional methods, including inactivated whole virus, live-attenuated virus and protein subunit of the virus, and next-generation techniques, including messenger RNA (mRNA), DNA and viral vector-based technologies 7-9. The minimal criterion for an acceptable Covid-19 vaccine based on WHO and FDA proposals is a clear demonstration of at least 50% vaccine efficacy. WHO suggested trial endpoints should assess disease, severe disease and/or shedding/transmission, while FDA suggested that laboratory confirmed Covid-19 or SARS-CoV-2 infections are both appropriate primary endpoints for vaccine efficacy 10.

A year after the start of the SARS-CoV-2 outbreak, there are 64 vaccine candidates against it under clinical development 11. There are different types of vaccines currently under Phase III clinical trial development for which the results have either published in peer-review journals or announced as press releases. These include two mRNA-based, two viral vector-based, and one protein subunit-based vaccine platforms 12, 13. So far, three of them have already obtained approval in the US and Europe. Vaccine development has occurred at an unprecedented speed, never heard of prior to Covid-19, thanks to the advancement in vaccine technology, such as mRNA vaccines that can be readily adapted to new pathogens 14. Other than these three vaccine candidates, other vaccine candidates have also been approved outside of the US and Europe. Some of them are now gearing up to meet the requirements from multiple Health Authorities and will be launched in near future 12.

**Figure 1:**
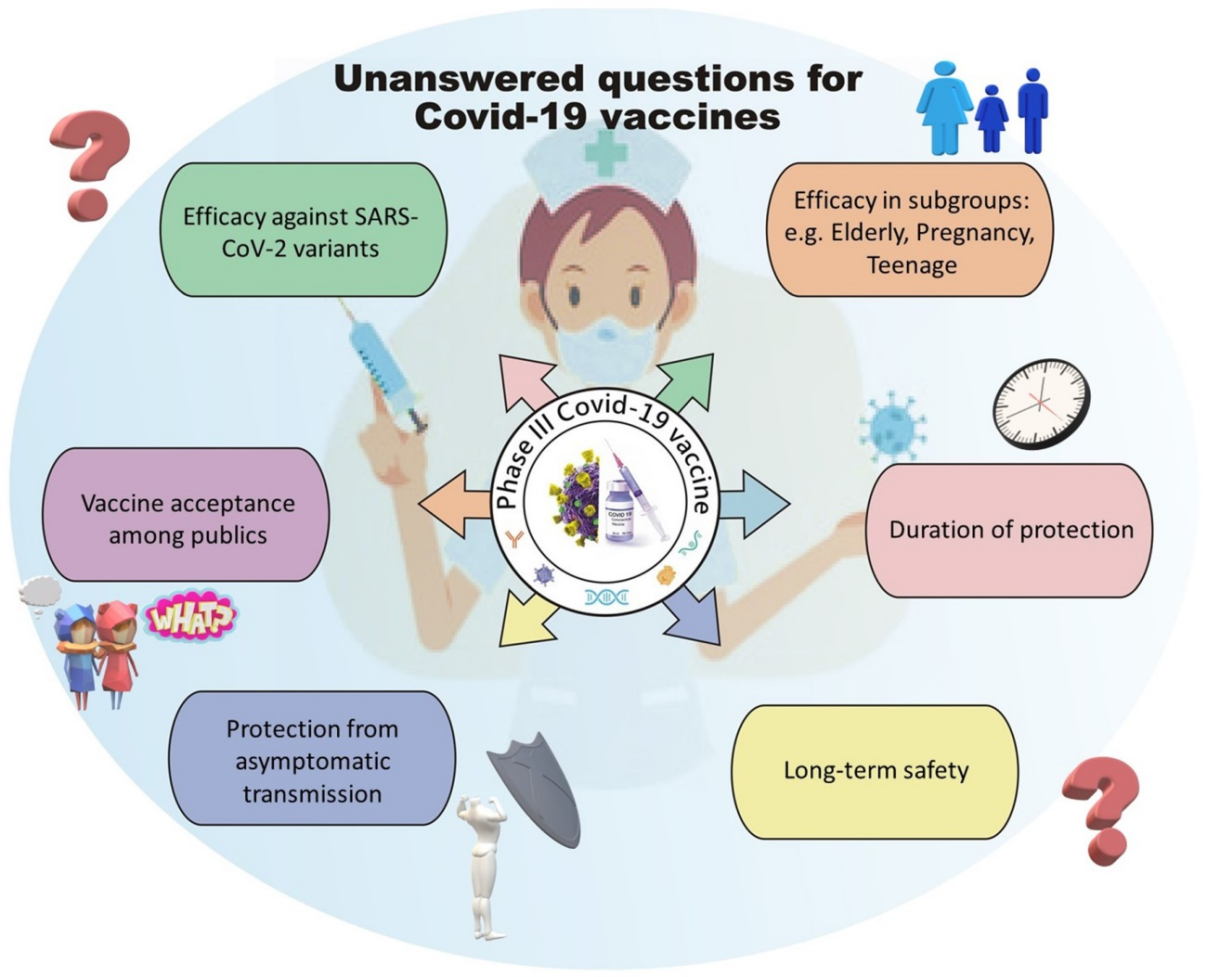
Current unanswered questions for Covid-19 vaccines

In a review paper Ebenezer Tumban has discussed what features constitute an ideal SARS-CoV-2 vaccine candidate to fight against the pandemic 15, but none of the vaccine candidates so far meet all the criteria listed. Moreover, additional criteria have emerged during the ongoing pandemic as we understand more about SARS-CoV-2, suggesting that even with current success in the Phase III clinical trial programs of various vaccine candidates, there are still many unanswered questions that required the scientific community to address before we can identify the ideal vaccine candidates for different conditions, boost public confidence and reduce vaccine hesitancy. These questions include 1) whether the vaccine candidate can elicit a long-lasting protective immune response and be proven safe in long-term follow up; 2) whether the vaccine can be given to people with various conditions, such as aging populations, adolescents, females of pregnant or breastfeeding status and other comorbidities; 3) whether the vaccine can elicit an immune response that protects individuals from various SARS-CoV-2 viral variants; 4) whether the vaccine can protect an individual from the asymptomatic transmission of SARS-CoV-2, hence protecting people ineligible for vaccination due to medical conditions by herd immunity; 5) whether the vaccine can be accessed by developing countries with affordable transportation and storage conditions and 6) whether the scientific community can provide confidence to the public to improve the rate of vaccination (Figure 1).

This review will discuss published pre-clinical and clinical data for several vaccine candidates and review any available data of these candidates in different human populations and conditions.

## Latest Covid-19 vaccines with Phase III Clinical Trials data released

### ChAdOx1 nCoV-19 adenoviral vector vaccine

ChAdOx1 nCoV-19 was developed at Oxford University and consists of a replication-deficient chimpanzee adenoviral vector containing the SARS-CoV-2 spike protein gene. It has been shown to elicit a robust humoral and cell-mediated response in mice and in rhesus macaques. Vaccination of ChAdOx1 nCoV-19 also protected rhesus macaques from SARS-CoV-2 induced pneumonia 16. One dose of this vaccine could induce antigen-specific antibody and T cell responses already, while the second dose of vaccination has shown to enhance the antibody responses and increase the SARS-CoV-2 neutralizing antibody in animal models 17. Similar results were seen in Phase I/II trial demonstrating that two doses of the vaccine could enhance anti-spike protein neutralizing antibody titers, Fc-mediated functional antibody responses, antibody-dependent neutrophil/monocyte phagocytosis, complement activation and natural killer cell activation, supporting the use of two doses in the later Phase of clinical trials 18. Another Phase I/II study showed that this vaccine has an acceptable safety profile and could elicit antibody responses in most participants 19. Similarly, the Phase II/III study also demonstrated that this vaccine is highly tolerable and can generate immunogenicity in most trial participants 20. An interim analysis of the efficacy and safety of ChAdOx1 nCoV-19 vaccine combining four clinical studies has also been published 21. The primary efficacy analysis includes 2 of the 4 clinical studies totaling of 11,636 participants, with 30 Covid-19 cases among 5,807 participants in the vaccine arm and 101 cases among 5,829 participants in the control arm, resulting in an overall vaccine efficacy of 70.4%. The vaccine had a good safety profile with serious adverse events and adverse events of special interest balanced across the study arm. Limitations of the study include the inclusion of younger age groups, and more white and female participants, which are typically a lower risk population for severe disease. The follow-up time of this study is still relatively short, with all disease episodes accrued within six months of the first dose being administered. Further investigation on this vaccine may focus on an intramuscular route of vaccination, which has been shown to reduce SARS-CoV-2 shedding and decrease viral load in lung tissues 22.

### BNT162b2 mRNA vaccine

Before COVID-19 pandemic, no mRNA drug or vaccine was licensed for use in humans. BNT162b2 is co-developed by BioNTech and Pfizer. It is a lipid nanoparticle-formulated nucleoside-modified RNA encoding the full-length spike protein. This vaccine candidate was selected for further clinical development among the four potential mRNA vaccine candidates based on the evidence of its ability to elicit neutralizing antibodies with lower incidence and severity of systemic reactions 23. The Phase III study consists of 43,548 participants, with 8 cases of Covid-19 identified among 17,411 participants in the vaccine arm and 162 Covid-19 cases identified among 17,511 participants in the control arm, resulting in a vaccine efficacy of 95% 24. Local reaction was generally mild-to-moderate in severity and resolved within 1 to 2 days, and no participant reported any grade 4 local reaction with the 2 doses of injection. Systemic events, including fever and chills, were observed within 1 to 2 days after vaccination and resolved shortly thereafter. More participants (21%) have reported treatment-related adverse events in the vaccine arm (21%) compared to the control arm (5%), and this increase could mostly be attributed to the inclusion of transient events. In the vaccine arm, only 4 vaccine-related serious adverse events were reported, and no deaths were considered by the investigators to be related to the vaccine (n = 2) or placebo (n = 4). The key limitations of this report are the short follow-up time, with a median follow-up of only two months after the second dose, and that the analysis has not addressed whether vaccination prevents asymptomatic infection. Based on the data, BNT162b2 became the first Covid-19 vaccine authorized for emergency use by the US FDA 25.

### mRNA-1273 mRNA vaccine

mRNA-1273 is co-developed by researchers at the National Institute of Allergy and Infectious Disease, and Moderna mRNA-1273 encodes the S-2P antigen consisting of the SARS-CoV-2 glycoprotein with a transmembrane anchor and an intact S1-S2 cleavage site. An animal study of mRNA-1273 has shown its robustness in inducing SARS-CoV-2 neutralizing activity, protecting upper and lower airways as well as the lung 26. In a Phase I study, mRNA-1273 was able to induce anti-SARS-CoV-2 immune responses in all participants with no trial limiting safety concern was identified 27. The safety profile of this vaccine was also tested in older adults, and there were mainly mild or moderate adverse events reported.^28^ The Phase III study consisted of 30,420 participants, with 185 cases of Covid-19 identified in the control arm and 11 cases in the vaccine arm, resulting in a 94.1% vaccine efficacy 29. Solicited adverse events at the injection site occurred more frequently in the vaccine arm compared to the control arm. Still, they were mainly Grade 1 or 2 in severity and lasted on average only 2-3 days after the first or the second dose. Solicited systemic adverse reactions occurred more frequently in the vaccine arm compared to the control arm, while the severity of the systemic events increased in the second dose compared to the first dose, but the events also lasted for an average of around only 2-3 days after the first or second doses. On the other hand, the frequency of unsolicited adverse events, severe adverse events and serious adverse events reported during the 28 days after injection were generally similar between participants in the vaccine and control arms. Similar to the previous two vaccine candidates, the one key limitation of the recently presented data is the short duration of safety and efficacy follow up time, with a median follow up time of only two months at the time of data cut off. There was also no evaluation of asymptomatic infection at the time of data reporting. Nonetheless, an update of the immunogenicity data from a Phase I trial showed that mRNA-1273 produced a high level of binding and neutralizing antibody responses. Although these responses declined slightly over time, all participants remained elevated at 90 days after the second vaccination in the 34 healthy adult participants tested. These results suggest that mRNA-1273 has the potential to provide durable humoral immunity 30.

### Ad26.COV2.S adenovirus vector vaccine

Ad26.COV2.S is developed by Janssen. It is a recombinant replication-incompetent adenovirus serotype 26 vectors encoding a full-length SARS-CoV-2 spike protein. The Phase I study has shown that the vaccine is safe, with only 5 out of 401 participants reported to have serious adverse events, while no participant discontinued the trial because of an adverse event. In addition, neutralizing antibody was detected in all participants suggesting that Ad26.COV2.S vaccination resulted in a high level of immunogenicity 31. Together, the Phase I study results support the entry of this vaccine into a Phase III study. The Phase III study consists of 44,325 participants; an interim analysis assessing 468 symptomatic Covid-19 cases showed that this single-dose vaccine had a vaccine efficacy of 66% in preventing moderate and severe Covid-19 at 28 days post-vaccination (Janssen Press Release: https://www.nih.gov/news-events/news-releases/janssen-investigational-covid-19-vaccine-interim-analysis-phase-3-clinical-data-released).

### NVX-CoV2373 protein subunit vaccine

NVX-CoV2373 is developed by Novavax and contains Matrix-M1 adjuvant and a recombinant SARS-CoV-2 full-length wild-type spike glycoprotein. The vaccine has been tested in various animal models; its ability to induce immunogenicity and provide protection against SARS-CoV-2 challenge in these animal models has been demonstrated 32, 33. In a Phase I study, the vaccine was shown to be safe and elicited appropriate immune responses 34. Follow up with a Phase III study in the UK has shown an overall vaccine efficacy of 89.3%, and 86% against the UK emerged variant B.1.1.7 (Novavax Press Release: https://www.novavax.com/sites/default/files/2021-01/UK-SouthAfrica-Trial-Results--FINAL.pdf).

### CoronaVac inactivated virus vaccine

CoronaVac is an inactivated virus vaccine developed by Sinovac, which elicits immune response directed against many antigens of the SARS-CoV-2 instead of targeting only the spike protein. The Phase III double-blind placebo-controlled clinical trial assessed this inactivated viral vaccine's efficacy and safety in healthcare professionals using an immunization schedule of two doses of intramuscular injections with a 14-day interval. Although the results of this Phase III study are yet to be published in a peer-reviewed journal, the Phase II data showed seroconversion of neutralizing antibodies higher than 97% with the incidence of adverse reactions of less than 35% 35. The company also announced their Phase III trial data achieving a vaccine efficacy of 51% for all cases, 84% for cases requiring medical treatment, and 100% for severe, fetal cases and cases requiring hospitalization (Sinovac Press Release: http://www.sinovac.com/?optionid=754&auto_id=922).

## Vaccine efficacy and safety in different populations

Vaccine efficacy and safety may differ in different human populations and also in different conditions, however, most of the Phase III clinical trials only assess vaccine efficacy and safety in a very well-defined human population and conditions, for example limiting the participants in a narrow age range, testing in only few countries, excluding participant with pregnancy status or other complicated medical conditions etc. This may result in difficulty generalizing the Phase III data into a broader population and some specific conditions. In view of this, many follow up trials have investigated specific human populations or conditions in order to elucidate whether the data can be upheld in such circumstances.

The elderly population has been affected the most in this Covid-19 pandemic, being not only more susceptible to SARS-CoV-2 infection, but also developing more severe life-threatening Covid-19 associated symptoms. While their immune response triggered by vaccination may differ from the younger population due to the decline of their immune system, comorbidities and pharmacological treatments, only a small fraction of the clinical studies tested the vaccine efficacy and safety in this subpopulation 36.

In the ChAdOx1 nCoV-19 Phase III trial,^21^ the majority (88%) of the participants included in the primary efficacy analysis were aged between 18 and 55 years. The efficacy in older age groups has not yet been assessed since there were only 5 Covid-19 cases reported in the primary analysis. In the BNT162b2 phase III study 24, the vaccine efficacy was consistent among different age subgroups. The vaccine efficacy was 95.6% in participants aged between 16 to 55 years, and 93.7%, 94.7% and 100% in participants aged above 55, above 65 and above 75 years, respectively. In the mRNA-1273 Phase III study 29, the vaccine efficacy for participants who aged between 18 to 65 was 95.6%, while for those aged higher than 65 the efficacy was 86.4%. A Phase I dose-escalation study of mRNA-1273 was expanded to include 40 older adults, 20 of which were in the age group of 56-70, while another 20 participants were older than 70 years. Adverse events associated with the vaccine were mainly mild or moderate with no serious adverse events reported, and there was only one participant that did not receive the second dose due to development of a maculopapular rash, though that was considered by investigators to be unrelated to vaccination. In this expansion study, the binding- and neutralizing-antibody responses appeared to be similar in these older adult participants, compared to those aged between 18 to 55 years, while the vaccine also elicited a strong CD4 cytokine response in this older population 28.

The results so far indicate that some vaccine candidates have acceptable efficacy and safety profile the elderly population, more clinical data focusing on this population is required to fully elucidate the benefit-risk ratio to inform healthcare professionals and to educate the public during the vaccination program, especially in areas with high vaccine hesitancy.

Another untapped age group in all these available Covid-19 vaccine Phase III clinical trial studies are pediatrics and adolescent populations. No children age under12 were enrolled in these Phase III clinical trials, while data from the 12 to 16-year-old subgroup have yet to be published. Lack of data in these populations will also result in vaccine hesitancy, especially among parents with younger kids, where they have milder symptoms of Covid-19 with SARS-CoV-2 infection. Having more data in pediatric and adolescent populations would a key to our ability in establishing herd immunity for SARS-CoV-2 37.

Some data are showing that Covid-19 may differentially impact patients of different ethnicity.^38^ Most of the published Phase III studies on Covid-19 consisted of a majority of white participants and very low rates of participation by other ethnicities, including black communities 38. In the ChAdOx1 nCoV-19 phase III trial 21, the majority (83%) of the participants included in the primary efficacy analysis were white, however, the results in different ethnicity groupings have not been discussed. In the BNT162b2 study 24, the vaccine efficacy for white participants was 95.2% while for ethnicities other than white it was 93.9%. In the mRNA-1273 study 29, the vaccine efficacy for white was 93.2% while for ethnicities other than white was 97.5%.

Vaccine efficacy and safety data specific for participants with comorbidities and specific conditions are limited. In the BNT162b2 study 24, the vaccine efficacy in participants with obesity was presented. The data demonstrated that similar vaccine efficacy was observed in the subgroup of participants with obesity (95.4%) and without obesity (94.8%), even taking age and obesity together for consideration, the vaccine efficacy was still above 90% in all of the subgroups. In the mRNA-1273 Phase III trial 29, the vaccine can also protect the population which is at risk for severe Covid-19. For participants who were at risk of severe Covid-19, 43 of them in the control arm and 4 in the vaccine arm developed Covid-19, resulting in an efficacy of 90.9%.

Vaccine efficacy and safety have not been reported in pregnant women in any clinical trials for any of the vaccine candidates. The only known information related to Covid-19 vaccine and pregnancy was specific to mRNA-1273 vaccine, where the emergency use authorization fact sheet mentioned that a reproductive toxicity study in female rats, where vaccine-related adverse effects on female fertility, fetal development, and postnatal development were evaluated with no adverse events being reported. A pregnancy exposure registry to monitor pregnancy outcome in woman vaccinated with mRNA-1273 has also been set up for data collection 39.

## Vaccine efficacy in protecting the development of severe Covid-19

In the ChAdOx1 nCoV-19 vaccine Phase III trial 21, the vaccine efficacy for asymptomatic infection was very low, with 71 cases detected among the 5,511 participants, 34 of them were in the vaccine arm while 37 were in the control arm, resulting in a vaccine efficacy of only 7.8%. This data indicated that ChAdOx1 nCoV-19 may not be an ideal vaccine candidate to stop asymptomatic transmission of SARS-CoV-2. In the same study, there were ten Covid-19 cases requiring hospitalization; all of them were from the control arm, with 2 of them developing severe Covid-19 including one fetal case, suggesting that ChAdOx1 nCoV-19 is effective in protecting participants from developing severe Covid-19. In the BNT162b2 study^24^, the asymptomatic infection has not been evaluated. Regarding protection against severe Covid-19, 9 out of 21,686 participants developed severe Covid-19 in the control arm while only 1 developed severe Covid-19 in the vaccine arm, resulting in an efficacy of 88.9%. In the mRNA-1273 Phase III trial 29, there were 30 participants that developed severe Covid-19 and all of them were in the control group, demonstrating a 100% vaccine efficacy against the development of severe Covid-19. Taken together, these results suggest that although there is uncertainty in whether these vaccine candidates effectively stop SARS-CoV-2 transmission, they are highly effective in diminishing the ability of SARS-CoV-2 in inducing severe symptoms upon infection.

## Vaccine efficacy and SARS-CoV-2 variants

With the recent surge in Covid-19 cases in both the UK and South Africa and the identification of new SARS-CoV-2 variants in these two countries, whether the tested vaccine candidates can protect us from these variants becomes an unanswered question, and raises public concerns on the efficacy of these vaccine candidates in the real world.

These recently emerged new variants are named B.1.1.7 in the UK and B.1.351 in South Africa. The UK variant B.1.1.7 contains the D614G mutation as well as eight other spike protein mutations. In comparison, the South Africa variant B.1.351 also contains the D614G mutation with nine other spike protein mutations 40. These two variants have been demonstrated *in vitro* to be refractory, at a different degree, to neutralization by a monoclonal antibody against spike protein and the receptor-binding domain, leading to uncertainty on the vaccine efficacy towards these newly emerged variants 40.

A recently published reported has shown that sera from 40 participants who were vaccinated with BNT162b2 had the ability to neutralize both the Wuhan reference strain and the B.1.1.7 lineage that has newly emerged in the UK, although to a lesser extent, suggesting that BNT162b2 can still induce protection against the B.1.1.7 lineage 41. Similarly, 20 human sera from the BNT162b2 trial also exhibited equivalent neutralizing titers to the N501 and Y501 virus, suggesting that BNT162b2 may produce protection also against the new viral strain that emerged from the UK and South Africa which contains a N501Y substitution 42.

Recent research has also tested the ability of mRNA-1273 vaccine in the induction of neutralizing antibodies against different spike mutants from SARS-CoV-2 variants. The study has tested six SARS-CoV-2 variants and shown no significant impact on neutralization against the B.1.1.7 variant that emerged from the UK. Still, a reduced neutralization was observed against the mutations present in the B.1.351 variant emergent from South Africa. Nevertheless, even with the decreased neutralization response, the sera from vaccinated individuals still maintained a relatively high level of neutralization ability 43.

On the other hand, another study has shown that the plasma of a cohort of 20 volunteers who received either mRNA-1273 or BNT162b2 vaccines had developed plasma neutralizing activity. However, the activity against SARS-CoV-2 variants encoding E484K or N501Y, or the K417N:E484K:N501Y combination, was reduced slightly, indicating a potential of loss of clinical vaccine efficacy. This may happen with cumulative mutations in the spike protein of SARS-CoV-2 with time, suggesting that mRNA vaccines may need to be updated periodically in order to avoid potential loss of clinical efficacy 44.

Although the results of Phase III data of the NVX-CoV2373 have yet to be published in a peer-review journal, the preliminary data shown in their press release suggested that this vaccine has a 86% vaccine efficacy towards the variant strain emergent from the UK. In addition, the Phase IIb trial of this vaccine took place as the South Africa 501Y.V2 escape mutant was found to be dominating the infection in South Africa, and the results showed that the overall vaccine efficacy was at a level of 49.4%. Sequencing data have confirmed that 93% of the SARS-CoV-2 infected individuals could be attributed to the 501Y.V2 escape variant from South Africa. These results indicate that NVX-CoV2373 may produce protection against the South Africa SARS-CoV-2 variant, but at a lesser extent compared to the original and United Kingdom strains (Novavax Press Release: https://www.novavax.com/sites/default/files/2021-01/UK-SouthAfrica-Trial-Results--FINAL.pdf).

## Conclusion

Approval of the Covid-19 vaccines is just the first step to our success in combating SARS-CoV-2, and it is paramount that the scientific community can convince the public to improve vaccine acceptance and reduce vaccine hesitancy, especially in some subgroups under-represented in Phase III clinical trials (Table 1). We need further evidence in such subgroups in order to be educated, and be able to confidently inform those subgroups on their benefit and risk of taking the vaccine. Besides, the public also worries about vaccine efficacy in protecting the vaccinated population towards asymptomatic infection, developing severe Covid-19 and infection by ever-mutating SARS-CoV-2 variants. More data and continuous effort are required to update the scientific community and the public in this ongoing pandemic for us to declare "victory" against this highly infectious virus.

## Figures and Tables

**Table 1:** Different Phase III Covid-19 Vaccines efficacies and safeties in different population and against different SARS-CoV-2 variants

Vaccines	Participants' Age	Efficacy	Efficacy with ethnicity	Efficacy with comorbidities	Efficacy in pregnant and breastfeeding women	Efficacy against various strain of SARS-CoV-2 variants	References
ChAdOx1 nCoV-19	18-55	70.4%	83% for Caucasian	Not reported	Not tested	Not reported	21
	Above 55						
BNT162b2	16-55	95.6%	95.2% for Caucasian 93.9% for Other than Caucasian	95.4% with obesity 94.8% without obesity	Not tested	Not reported	24
	Above 55	93.7%					
	Above 65	94.7%					
	Above 75	100%					
mRNA-1273	18 to 65	95.6%	93.2% for Caucasian 97.5% for Other than Caucasian	Not reported	Not tested	Not reported	29
	Above 65	86.4%					
Ad26. COV2.S	18 and older	85%	Not reported	Not reported	Not tested	Not reported	https://www.nih.gov/new s-events/news- releases/janssen- investigational-covid-19- vaccine-interim-analysis- phase-3-clinical-data- released
NVX-CoV2373	18-65	89.3%	Not reported	Not reported	Not tested	86% efficacy against UK variant strain 49.4% efficacy against SA variant strain	https://www.novavax.co m/sites/default/files/2021 -01/UK-SouthAfrica- Trial-Results-- FINAL.pdf
	65 and above						
CoronaVac	18 and older	50.7%	Not reported	Not reported	Not tested	Not reported	http://www.sinovac.com/?optionid=754&auto_id=922

*UK = United Kingdom; SA= South Africa
